# A Study of the Head during Prenatal and Perinatal Development of Two Fetuses and One Newborn Striped Dolphin (*Stenella coeruleoalba*, Meyen 1833) Using Dissections, Sectional Anatomy, CT, and MRI: Anatomical and Functional Implications in Cetaceans and Terrestrial Mammals

**DOI:** 10.3390/ani9121139

**Published:** 2019-12-13

**Authors:** Álvaro García de los Ríos y Loshuertos, Alberto Arencibia Espinosa, Marta Soler Laguía, Francisco Gil Cano, Francisco Martínez Gomariz, Alfredo López Fernández, Gregorio Ramírez Zarzosa

**Affiliations:** 1 Departamento de Anatomía y Anatomía Patológica Comparadas, Facultad de Veterinaria, Universidad de Murcia, 30100 Murcia, Spain; 2 Departamento de Morfología. Anatomía y Embriología, Facultad de Veterinaria, Universidad de Las Palmas de Gran Canaria, Trasmontaña, Arucas, 35416 Las Palmas de Gran Canaria, Spain; 3 Departamento de Medicina y Cirugía, Facultad de Veterinaria, Universidad de Murcia, 30100 Murcia, Spain; 4 Departamento de Biología—CESAM, Universidade de Aveiro, Campus Universitario de Santiago, 3810-193 Aveiro, Portugal

**Keywords:** striped dolphin (*Stenella coeruleoalba*), fetal development, PET/SPECT/CT, MRI, sectional anatomy, head anatomy, ontogenesis

## Abstract

**Simple Summary:**

The head region of the dolphin has been studied widely to identify its anatomical structures and to compare it with other marine and terrestrial mammals. In this study, specimens stranded off the Spanish coast were used. Our study analyzes four dolphin heads during fetal and perinatal development. All specimens were scanned using modern imaging techniques to study their internal organs and to preserve the specimens, which are difficult to obtain. Only one fetus was transversely cross-sectioned to help us to identify critical organs. The developmental study shows several anatomical structures that are compared with cetaceans and terrestrial mammals. During development of the oral cavity, it was observed that the rostral maxillary and mandible teeth (incisive area) had not completely erupted, in contrast with the rest of teeth, which have done so. Also, the main chewing muscle (masseter) was not observed. In addition, we describe the absence of major salivary glands during these developmental stages. Furthermore, we explain the characteristics of the orbit and its relation to the eyeball. In addition, the fetal dolphin’s ear is connected with pharynx in a way similar to that in horses. We conclude that these developmental studies will help cetacean conservation.

**Abstract:**

Our objective was to analyze the main anatomical structures of the dolphin head during its developmental stages. Most dolphin studies use only one fetal specimen due to the difficulty in obtaining these materials. Magnetic resonance imaging (MRI) and computed tomography (CT) of two fetuses (younger and older) and a perinatal specimen cadaver of striped dolphins were scanned. Only the older fetus was frozen and then was transversely cross-sectioned. In addition, gross dissections of the head were made on a perinatal and an adult specimen. In the oral cavity, only the mandible and maxilla teeth have started to erupt, while the most rostral teeth have not yet erupted. No salivary glands and masseter muscle were observed. The melon was well identified in CT/MRI images at early stages of development. CT and MRI images allowed observation of the maxillary sinus. The orbit and eyeball were analyzed and the absence of infraorbital rim together with the temporal process of the zygomatic bone holding periorbit were described. An enlarged auditory tube was identified using anatomical sections, CT, and MRI. We also compare the dolphin head anatomy with some mammals, trying to underline the anatomical and physiological changes and explain them from an ontogenic point of view.

## 1. Introduction

Cetaceans are a group of mammals well adapted to their marine environment and whose evolutionary changes are especially marked in the development of the structures of the head. In both suborders of living cetaceans, the skull has been highly modified by changes in feeding apparatus and the elimination or reduction of many structures [1]. The relationship of the bones in the skull to one another is altered due to the caudal migration of the nasal opening, a process known as telescoping [2,3,4]. In addition, differences occur in the location of the external nasal passages and the structure of the middle and the inner ear.

The study of an extensive collection of embryos and fetuses of these species has produced valuable information about the ontogeny of most of the body systems and about musculoskeletal development. Comparisons with other mammals detected the time lag in ossification, retardation of odontogeny, and the origin and development of the fluke, dorsal fin, and flipper [5].

Nevertheless, the studies performed so far lack information on the prenatal and perinatal development due to the difficulty in establishing the differences in ontogenetic development of cetaceans [6]. The precise time intervals of such development and any distinctive growth trajectories are basically unknown [7].

Even now, studies of cranial anatomy by anatomical sections seem to be scarce, with most studies performed in odontocetes (due to their smaller size) either in adults, for instance in common and striped dolphins [8,9], or in newborn bottlenose dolphins [10,11], pacific spotted dolphins, common dolphins, and narwhals [12], and in the fetal narwhal, common dolphin, Atlantic white-sided dolphin [13,14,15], and Beaked whale [16]. Fetal studies are least common due to the lack of stranded pregnant females. In the case of mysticetes, the few existent studies are focused almost exclusively on external anatomy: eye, nose, hair, and throat of a neonate gray whale [2]; osteology: skull anatomy in fetal specimens of whales of the genera *Megaptera* and *Balaenoptera* [17]; musculoskeletal: musculoskeletal anatomy of the head of a neonate gray whale [18] or vascular [18]. An exception to this is the work of Schute [19] in which a monograph study of the fetal anatomy of the Sei whale (*Balaenoptera borealis*) was done.

In both orders, outlines of organs are observed during the embryonic period and the organs are almost developed or in a development phase in the fetal period, which is of key importance as this is almost the only different period compared to the adult stage, because, for survival reasons, cetaceans give birth to precocial newborn. During these stages (end of the fetal and all the perinatal period), we can obtain valuable data on the species’ ontogeny, so we concur with [7] about the many applications in the fields of biology and animal medicine. One of these applications could be determining an organ’s development during the fetal period, helping researchers to calculate approximately the time of fetal development in odontocetes based on anatomical changes during gestation, similar to the Carnegie system designed for the human fetus [20] or for terrestrial mammals [3]. So far, we can only estimate cetacean parameters such as the gestation time by using a mathematical formula in *Stenella longirostris* [21], calculate the time of parturition using ultrasonography in Bottlenose dolphins [22], or estimate the adult’s age through dental growing lines in striped dolphins [23].

In the current study, we analyze the head anatomy of two striped dolphins’ (*Stenella coreuleoalba*) fetuses and one newborn of the same species. In each case, anatomical sections were correlated with computed tomography (CT) and magnetic resonance imaging (MRI).

Our goal is to accomplish several objectives: (a) to create a cephalic anatomy atlas of images during the fetal period up to the perinatal period, which could have benefits for cetacean conservation; (b) to collaborate with other studies dealing with the chronology of fetal development of these species; (c) to clarify some functional aspects of the anatomical structures of the head during prenatal and perinatal dolphin development; and (d) to accurately describe the structures of the head following the Illustrated Veterinary Anatomical Nomenclature [24].

## 2. Materials and Methods

### 2.1. Animals

A total of four pre- and perinatal specimens and one adult striped dolphin (*Stenella coeruleoalba*, Meyen 1833) were used in this study (Table 1). The mother of the youngest fetus was stranded on the Spanish Atlantic coast. The mother of the older fetus and two newborn specimens were stranded on the Spanish African coast. The adult specimen was stranded on the Spanish Mediterranean coast. Stranded specimens were found dead and ethics committee clearance was not necessary. Both fetuses and the newborn specimen were transported to the CT and MRI units to perform CT and MRI scans.

### 2.2. Computed Tomography

The sco1 was scanned with Positron Emission Tomography (PET), Single Photon Emission Computed Tomography (SPECT)-Computed Tomography (CT) (PET/SPECT/CT Albira^TM^ Systems, Valencia, Spain; Centro de Investigación Biomédica, Universidad de Murcia, Spain); single-slice: 1 detector arrays; type of acquisition: helical; thickness: 0.125 mm; image reconstruction interval or index: 0.0125 mm; pitch: 0; tube rotation time: 0.12; mA: 0.4; Kv: 45; FOV 68 cm, Matrix dimensions 2240 × 2360; reconstruction algorithm: FBP filtered back projection; WW: 600/WL: 300). The SPECT-CT images were transferred to a Dicom workstation, while sco2 was scanned with CT (General Electric Medical Systems, Schenectady, NY, USA; Clínica Virgen de Africa, Ceuta, Spain); multislice: 4 detector arrays; type of acquisition: helical; thickness: 5 mm; index: 3.2 mm; pitch: 0.45; tube rotation time: 0.33; mA: 30; kV: 120; FOV 35 cm; matrix dimensions: 512 × 512, reconstruction algorithm: bone; WW: 350/WL: 221; WW 650/WL −34. Finally, sco3 was scanned with a CT (General Electric Medical Systems-HiSpeed dual, Schenectady, NA, USA; Hospital Clínico Veterinario, Universidad de Murcia, Spain); multislice: 2 detector arrays; type of acquisition: helical; thickness: 5 mm; index: 2.5 mm; pitch: 0.35; tube rotation time: 1; mA: 100; Kv: 120; Image field of view FOV 40 cm; matrix dimensions: 512 × 512, reconstruction algorithm: standard; WW: 350/WL: 221; WW 650/WL −34). All dolphin specimens were positioned in ventral recumbency. All CT images were transferred to a DICOM workstation and CT images were analyzed with Radiant DICOM viewer and Osiris 4.0 for Windows. A vascular window setting (WW 600/WL 300) was applied to obtain PET/SPECT/CT images. Mediastinum-vascular window (WW 350/WL 221) and soft-tissue window settings (WW 650/WL −34) were applied to obtain Ceuta and Murcia CT images, respectively.

### 2.3. Magnetic Resonance Imaging

In sco1, Magnetic Resonance (MR) images were obtained with a high-field MR apparatus (General Electric Sigma Excite, Schenectady, NA, USA; Centro Veterinario de Diagnóstico por Imagen de Levante, Ciudad Quesada, Alicante, Spain), 1.5 Tesla using a human wrist coil. T1-weighted spin eco (SE) and T2-weighted fast spin scho (FSE) pulse sequences were used. T1-weighted (SE) images were obtained in transverse plane and 2D acquisition, using the following parameters: TE 13 ms, TR 640 ms, TI 0, NEX 1, slices thickness 1 mm, interslice gap 1.3, field of view 75 and matrix dimensions 0\256\192\0. T2-weighted (FSE) images were obtained in transverse plane and 2D acquisition, using the following parameters: TE 84 ms, TR 8100 ms, TI 0, NEX 1, slice thickness 1 mm, interslice gap 1.3, field of view 60, and matrix dimensions 192\0\0\192.

In sco2, MR images were obtained with a high-field apparatus (Philips Medical System Intera, Eindhoven, The Netherlands; Clínica Radiológica, Ceuta, Spain), 1.5 Tesla using a sense-body coil. T1-weighted fast field echo (FFE) and T1-weighted out-of-phase (OOP) gradient echo (GRE) pulse sequences were used. T1-weighted (FFE) images were obtained in transverse plane and 2D acquisition using the following parameters: TE 4.6 ms, TR 183 ms, TI 6, NEX 6, slice thickness 8 mm, interslice gap 9, field of view 68.6, matrix dimensions 0\204\155\0. T1-weighted (OOP) images were obtained in transverse plane and 2D acquisition using the following parameters: TE 2.3 ms, TR 130.3 ms, TI 0, NEX 5, 9 mm slice thickness, interslice gap 10, field of view 69.6, and matrix dimensions 132\0\0\103.

In sco3, MR images were obtained with a high-field apparatus (General Electric Sigma Excite, Schenectady, USA; Centro Veterinario de Diagnóstico por Imagen de Levante, Ciudad Quesada, Alicante, Spain), 1.5 Tesla using a human head coil. T1-weighted spin echo (SE) and T2-weighted fast spin echo (FSE) pulse sequences were used. T1-weighted (SE) images were obtained in transverse plane and 2D acquisition using the following parameters: TE 11 ms, TR 640 ms, TI 0, NEX 1, slice thickness 4 mm, interslice gap 4.5, field of view 75, and matrix dimensions 0\192\192\0. T2-weighted (FSE) images were obtained in transverse plane and 2D acquisition using the following parameters: TE 93.8 ms, TR 6020 ms, TI 0, NEX 1, slice thickness 4 mm, interslice gap 4.5, field of view 75, and matrix dimensions 0\192\192\0. All dolphin specimens were positioned in ventral recumbency. The MR images were transferred to a DICOM workstation. MR images were analyzed with Radiant DICOM viewer and Osiris 4.0 for Windows.

### 2.4. Anatomic Evaluation

Sco1, sco3, and sco4 were preserved by immersion in formaldehyde (10%). Sco5 was fixed with embalming solution (formaldehyde, glycerine, isopropyl alcohol, phenol) injecting the right and left carotid arteries and left and right external jugular veins. After 48 h, the carotid arteries and jugular veins were injected with red and blue latex, respectively. These specimens were stored in the Department of Anatomy and Embryology’s freezer chamber, Facultad de Veterinaria, Murcia, Spain. Sco3 was preserved frozen (−20 °C) in the Department of Anatomy and Embryology’s cooling chamber, Facultad de Veterinaria, Murcia, Spain.

Sco2 was frozen at −80 °C and then taken out to obtain cross sections cut with a band saw (Anatomical Lab, Department of Anatomy and Embryology, Universidad de Murcia, Murcia, Spain), obtaining 0.7–1 cm thick slices, which were then photographed giving us 57 transverse images used to correlate the sections with CT and MR images. Slices were immersed in acetone for plastination preservation and then stored in a freezer chamber at the Department of Anatomy and Embryology, Facultad de Veterinaria, Universidad de Murcia, Spain.

### 2.5. Gross Dissections

A deep head dissection of sco4 showed the melon. At its midpoint, the melon was cut in transverse and horizontal sections, which showed the nucleus and peripheral tissue ring. The nasal plug and nasal cavity were observed after removing the nasal vestibule and spiracle.

The head, face, and adjacent areas of sco5 were superficially dissected showing frontal and facial fat, the melon surface, and the mandible and superficial facial muscles. After carefully removing superficial fat and fibrous tissues, the venous drainage, several depressor mandible muscles, tongue muscles, and the rudiment of external acoustic meatus were exposed.

## 3. Results

### 3.1. Oral Cavity

**Figure 1:**
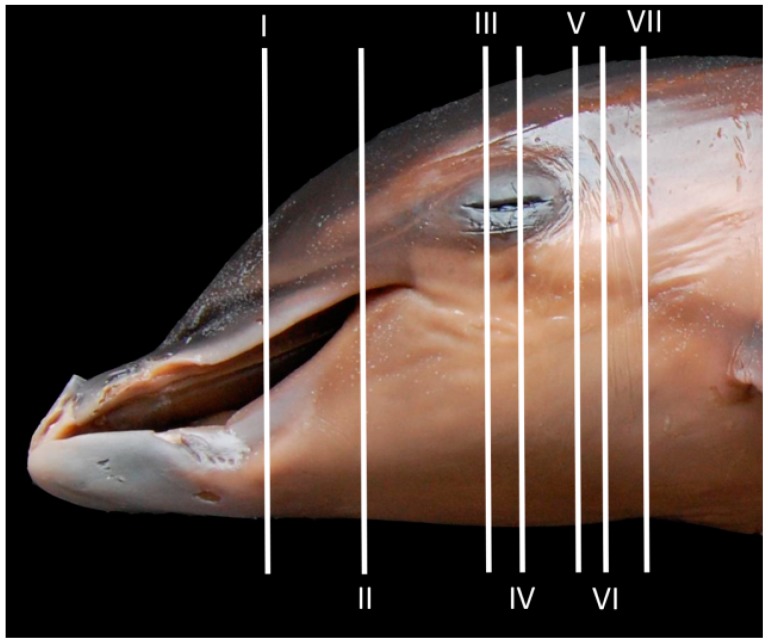
Approximated level sections of fetus dolphin head. Lines represent the location for each transverse anatomical section, CT, and MR images (I–VII).

**Figure 2:**
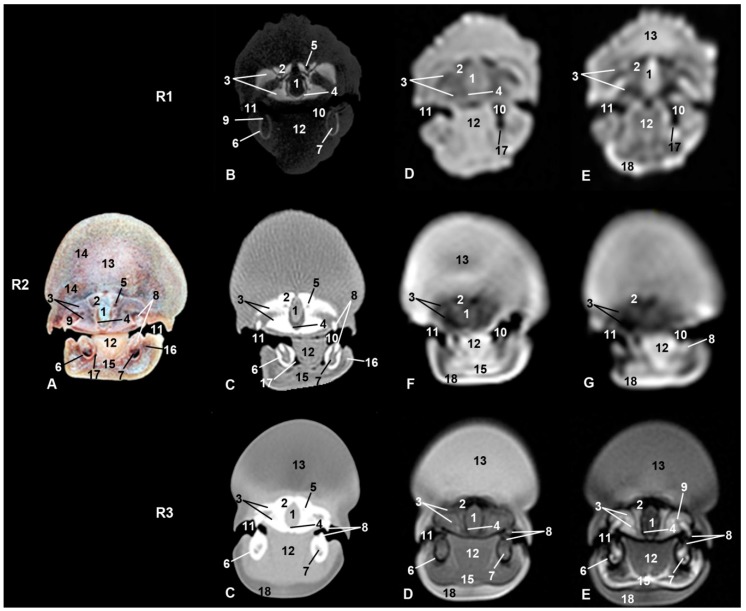
Representative transverse images of the snout made at the level of the rostral portion of the melon and oral cavity. Level I. Images are oriented so that the left side of the head is to the right and dorsal is at the top. Row (from now on R) 1, sco1; R2, sco2; R3, sco3. (**A**) Anatomical section. (**B**) Vascular window PET/SPECT/CT image. (**C**) Soft-tissue window CT image. (**D**) T1-weighted SE sequence. (**E**) T2-weighted fast spin echo (FSE) sequence. (**F**) T1-weighted fast field echo (FFE) sequence. (**G**) T1-weighted out of phase (OOP) gradient echo (GRE) sequence. 1, Mesethmoid cartilage; 2, incisive bone; 3, maxillary bone; 4, vomer bone; 5, supraorbital canal; 6, mandible; 7, canal and mandibular fat; 8, tooth in development; 9, socket of tooth; 10, oral cavity; 11, oral vestibule; 12, tongue; 13, melon; 14, melon rostral muscles; 15, mylohyoid muscle; 16, buccinator and depressor of the lower lip muscles; 17, lateral sublingual recess; 18, epidermis and dermis.

**Figure 3:**
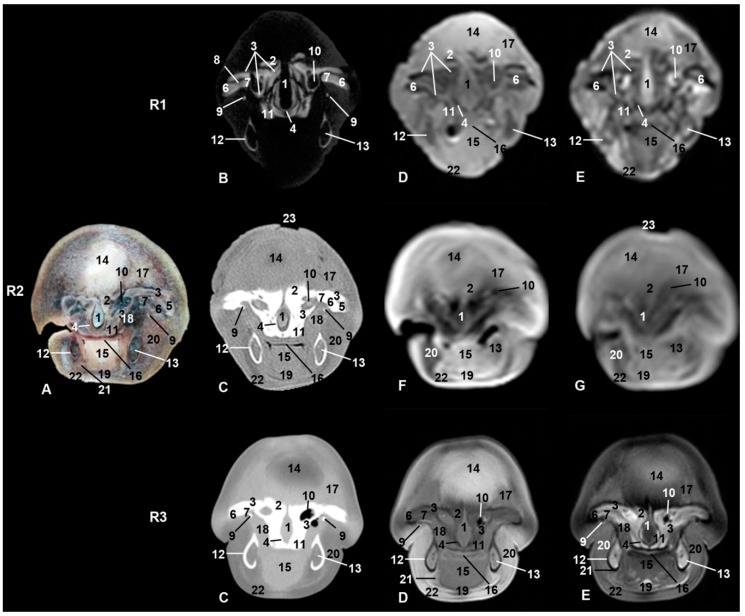
Representative transverse images made at the level of root of the snout, caudal portion of the melon, and oral cavity. Level II. Images are oriented so that the left side of the head is to the right and dorsal is at the top. R1, sco1; R2, sco2; R3, sco3. (**A**) Anatomical section. (**B**) Vascular window PET/SPECT/CT image. (**C**) Soft-tissue window CT image. (**D**) T1-weighted SE sequence. (**E**) T2-weighted FSE sequence. (**F**) T1-weighted FFE sequence. (**G**) T1-weighted OOP GRE sequence. 1, Mesethmoid cartilage; 2, incisive bone; 3, maxillary bone; 4, vomer bone; 5, frontal bone; 6, lacrimal bone; 7, zygomatic bone; 8, lacrimal-zygomatic synchondrosis; 9, zygomatic bone: temporal process; 10, maxillary sinus; 11, palatine bone; 12, mandible; 13, canal and mandibular fat; 14, melon; 15, tongue; 16, oral cavity; 17, melon rostral muscles; 18, pterygoid muscles; 19, mylohyoid muscle; 20, digastric muscle; 21, fat and striated muscle; 22, epidermis, dermis and subcutaneous tissue; 23, nostrils.

**Figure 4:**
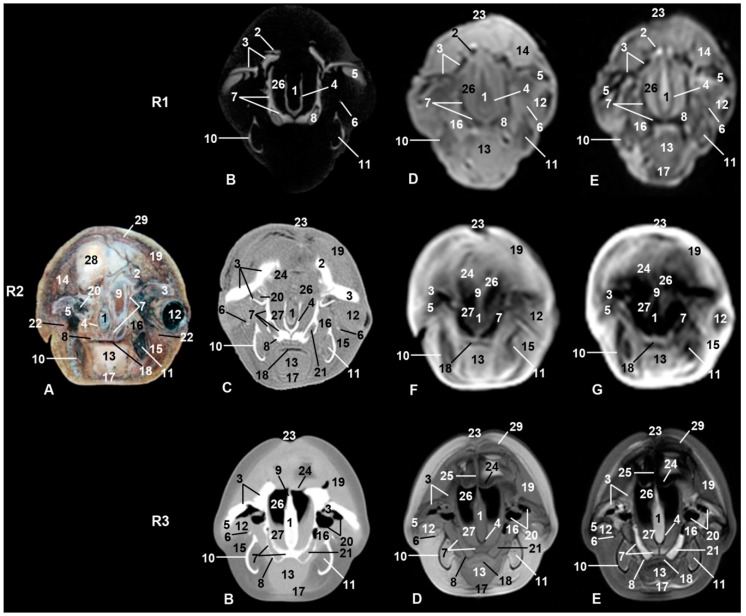
Representative transverse images made at the level of nasal, oral cavities, and orbital craniofacial fossa. Level III. Images are oriented so that the left side of the head is to the right and dorsal is at the top. R1, sco1; R2, sco2; R3, sco3. (**A**) Anatomical section. (**B**) Vascular window PET/SPECT/CT image. (**C**) Soft-tissue window CT image. (**D**) T1-weighted SE sequence. (**E**) T2-weighted FSE sequence. (**F**) T1-weighted FFE sequence. (**G**) T1-weighted OOP GRE sequence. 1, Mesethmoid cartilage; 2, incisive bone; 3, maxillary bone; 4, vomer bone; 5, lacrimal bone; 6, zygomatic bone: temporal process; 7, palatine bone; 8, pterygoid bone; 9, ethmoid bone; 10, mandible; 11, canal and mandibular fat; 12, periorbit and eyeball; 13, tongue; 14, melon external fiber ring; 15, digastric muscle; 16, pterygoid muscle; 17, mylohyoid muscle; 18, oral cavity; 19, melon caudal muscles; 20, frontal bone: orbital recess; 21, pterygopalatine recess; 22, fat and striated muscle; 23, nostrils; 24, nasal diverticulum and nasal plug (arrow); 25, membranous part of nasal septum; 26, nasal cavity; 27, nasal mucosa; 28, melon; 29, nasal vestibule muscles.

**Figure 5:**
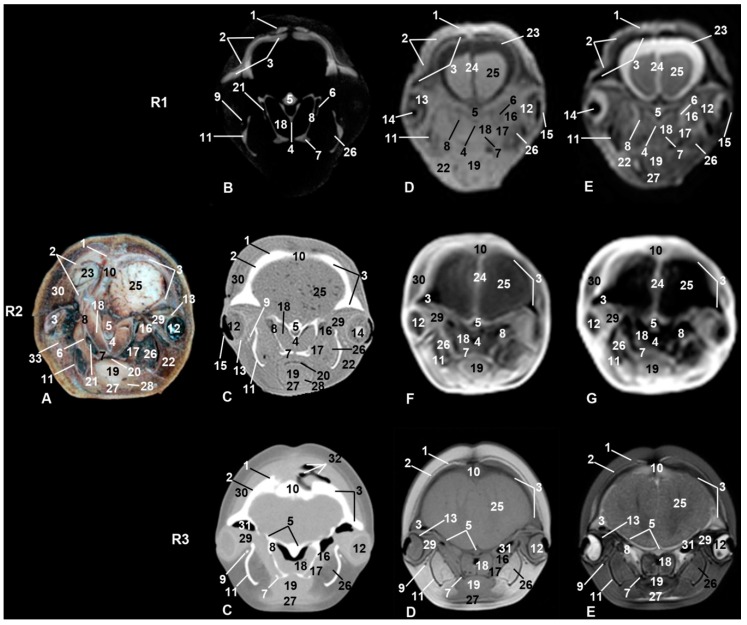
Representative transverse images made at the level of the rostral part of the cranial cavity, choanas, and eyeball. Level IV. Images are oriented so that the left side of the head is to the right and dorsal is at the top. R1, sco1; R2, sco2; R3, sco3; (**A**) Anatomical section. (**B**) Vascular window PET/SPECT/CT image. (**C**) Soft-tissue window CT image. (**D**) T1-weighted SE sequence. (**E**) T2-weighted FSE sequence. (**F**) T1-weighted FFE sequence. (**G**) T1-weighted OOP GRE sequence. 1, Incisive bone; 2, maxillary bone; 3, frontal bone; 4, vomer bone; 5, presphenoid bone: body and wings; 6, palatine bone; 7, pterygoid bone; 8, basisphenoid bone: pterygoid crest; 9, zygomatic bone: temporal process; 10, ethmoid bone; 11, mandible; 12, eyeball; 13, sclera; 14, lens; 15, eyelids; 16, lateral pterygoid muscle; 17, medial pterygoid muscle; 18, choanae and nasopharyngeal sphincter muscle; 19, tongue; 20, oral cavity; 21, pterygopalatine recess; 22, digastric muscle; 23, subarachnoid space; 24, longitudinal brain fissure; 25, brain: frontal lobe; 26, mandibular canal; 27, mylohyoid muscle; 28, hyoglossus muscle; 29, extraocular muscles; 30, melon caudal muscles; 31, frontal bone: orbital recess; 32, nasal diverticulum; 33, fat and striated muscle.

The oral cavity of the three studied specimens clearly showed the tongue in anatomical sections, CT, and MRI (Figure 1, Figure 2, Figure 3, Figure 4 and Figure 5). In sco1, the lateral sublingual recesses were observed only in MRI (Figure 2 Row (from now on R)(R1D-E)) while in sco2 it was identified in CT and anatomical sections Figure 2(R2A–C)). Under the lateral sublingual recess, it was not possible to distinguish sublingual salivary glands (neither polystomatic nor monostomatic). Histological analysis of tissue from this region showed a mixture of adipose and striated muscular tissue.

In sco1, CT showed clearly the dental alveolus dorsal to the mandibular canal but not in the maxillary bone (Figure 2(R1B)), while in sco2, CT and anatomical sections showed the most caudal teeth growing covered by gums in both the mandible and maxillary bones (Figure 2(R2C)). In the CT and MR images of sco3, the mid caudal teeth were forming in both dentary arches. In the three specimens, rostral maxillary and mandible teeth (incisive area) had not completely erupted, whereas the rest of mandible and maxillary teeth have done so in sco3 (Figure 2(R3C)).

**Figure 6:**
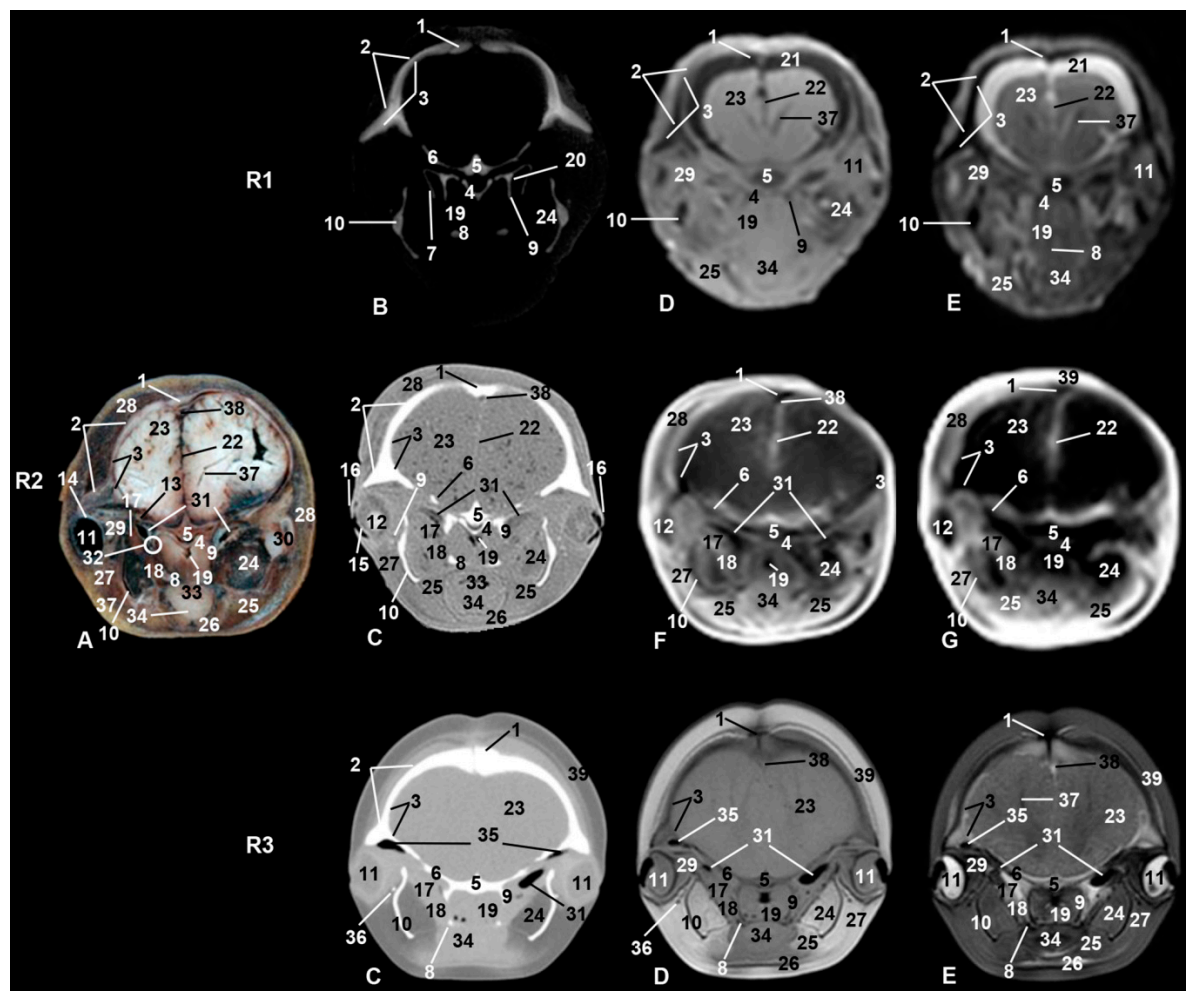
Representative transverse images made at the level of the pharynx and the caudal part of the orbit. Level V. Images are oriented so that the left side of the head is to the right and dorsal is at the top. R1, sco1; R2, sco2; R3, sco3. (**A**) Anatomical section. (**B**) Vascular window PET/SPECT/CT image. (**C**) Soft-tissue window CT image. (**D**) T1-weighted SE sequence. (**E**) T2-weighted FSE sequence. (**F**) T1-weighted FFE sequence. (**G**) T1-weighted OOP GRE sequence. 1, Nasal bone; 2, maxillary bone; 3, frontal bone; 4, vomer bone; 5, presphenoid bone: body; 6, presphenoid bone: wings; 7, palatine bone; 8, pterygoid bone: hook-like process; 9, pterygoid bone: pterygoid crest; 10, mandible; 11, eyeball; 12, lens; 13, optic nerve; 14, sclera; 15, cornea; 16, eyelids; 17, lateral pterygoid muscle; 18, medial pterygoid muscle; 19, nasopharynx and nasopharyngeal sphincter muscle; 20, pterygopalatine fossa; 21, subarachnoid space; 22, longitudinal brain fissure; 23, brain: temporal lobe; 24, mandibular canal; 25, digastric muscle; 26, sternohyoid muscle; 27, fat and striated muscle; 28, melon caudal muscles; 29, extraocular muscles; 30, temporomandibular joint; 31, auditory tube; 32, pharyngeal opening of the auditory tube; 33, oropharynx; 34, tongue; 35, frontal bone: orbital recess; 36, zygomatic bone: temporal process; 37, brain: lateral ventricle; 38, sagittal dorsal sinus; 39, epidermis, dermis, and subcutaneous tissue.

**Figure 7:**
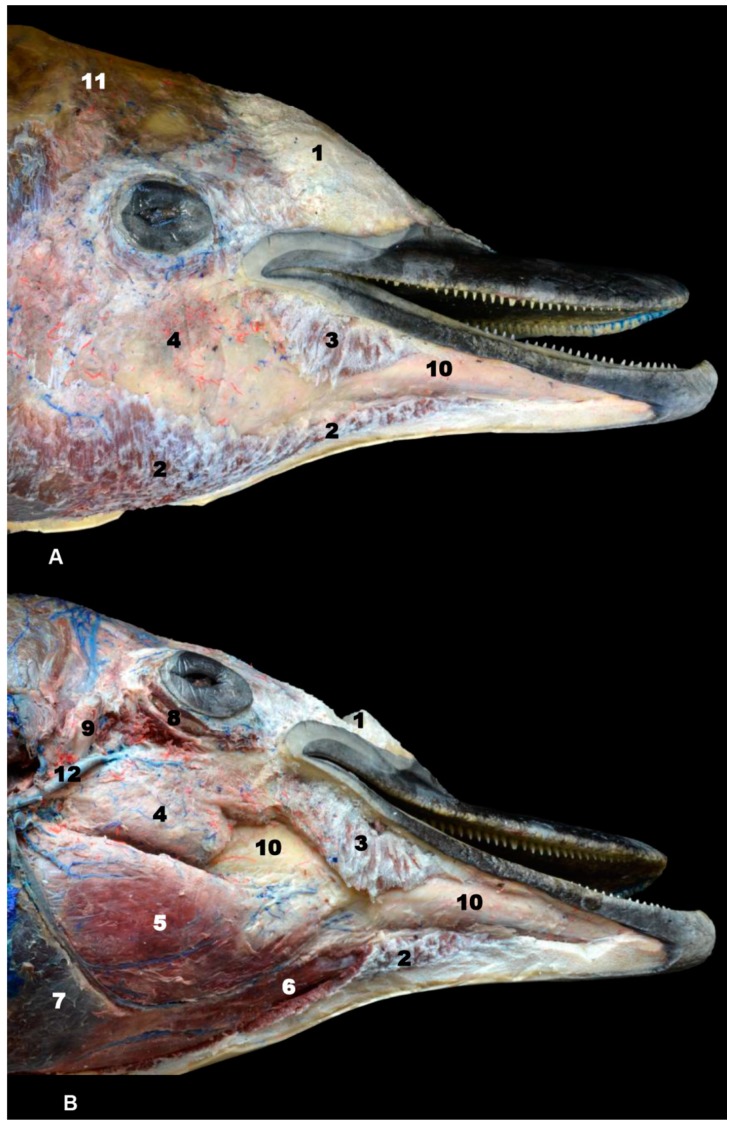
(**A**) Superficial and (**B**) middle head dissection made after removing melon and fat of sco5. 1, Melon; 2, mylohyoid muscle; 3, buccinator and depressor of the lower lip muscles; 4, fat and striated muscle; 5, digastric muscle; 6, geniohyoid muscle; 7, sternohyoid and sternothyroid muscles; 8, orbicularis oculi muscle; 9, external acoustic meatus (cartilaginous); 10, mandible: body; 11, subcutaneous tissue; 12, maxillary vein.

**Figure 8:**
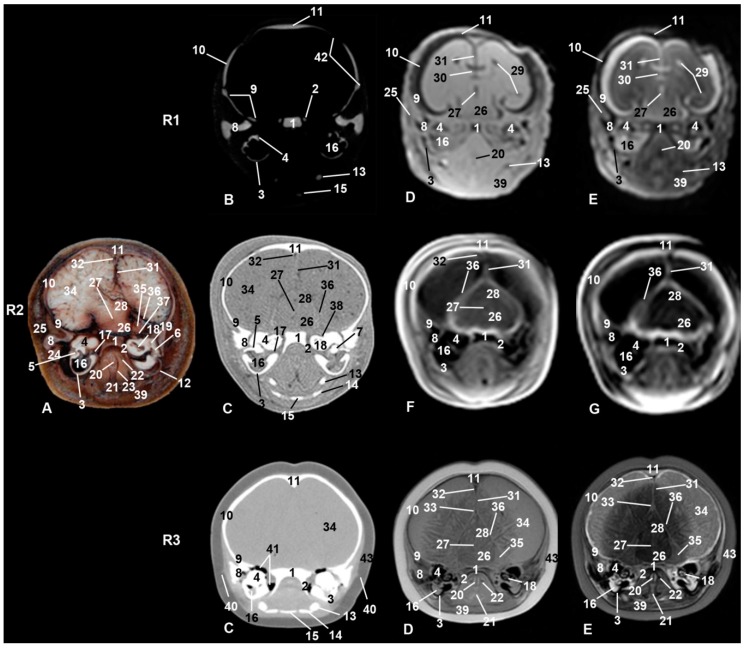
Representative transverse images at the level of the cranial vault of the skull involving the temporal lobe of the brain, mesencephalon, middle and inner ear, larynx and hyoid apparatus. Level VI. Images are oriented so that the left side of the head is to the right and dorsal is at the top. R1, sco1; R2, sco2; R3, sco3. (**A**) Anatomical section. (**B**) Vascular window PET/SPECT/CT image. (**C**) Soft-tissue window CT image. (**D**) T1-weighted SE sequence. (**E**) T2-weighted FSE sequence. (**F**) T1-weighted FFE sequence. (**G**) T1-weighted OOP GRE sequence. 1, Occipital bone: basilar part; 2, pterygoid crest; 3, temporal bone: tympanic part; 4, temporal bone: petrous part; 5, auditory ossicles of middle ear (malleus and incus); 6, auditory ossicles of middle ear (stapes); 7, auditory ossicles of middle ear (incus); 8, frontal process of temporal bone; 9, squamous part of temporal bone; 10, parietal bone; 11, interparietal bone; 12, tympanohyoid cartilage; 13, stylohyoid bone; 14, thyrohyoid bone; 15, basihyoid bone; 16, middle ear: tympanic cavity; 17, middle ear: musculotubarius canal; 18, inner ear: cochlea (spiral canal); 19, inner ear: vestibule; 20, arytenoid cartilage; 21, epyglotic cartilage; 22, nasopharynx: intrapharyngeal orifice; 23, laryngopharynx: piriform recess; 24, ramus of the mandible: condylar process; 25, temporal muscle; 26, mesencephalon: tegmentum; 27, mesencephalon: aqueduct; 28, mesencephalon: colliculus; 29, lateral ventricles; 30, corpus callosum; 31, falx cerebri; 32, dorsal sagittal sinus; 33, sinus transversus; 34, brain hemisphere: temporal lobe; 35, cerebellum: cerebellar hemispheres; 36, meninx: cerebellar tentorium; 37, facial and vestibulocochlear nerves and labyrinthic artery; 38, orifice and internal acoustic meatus; 39, sternohyoid muscle; 40, external acoustic meatus: cartilaginous rudiment; 41, peribullar sinus; 42, fontanelles; 43, epidermis, dermis, and subcutaneous tissue.

Only two of the three pairs of muscles of mastication were identified: temporal and pterygoid (Figure 4). The third, the masseter muscle, originates from the facial crest or maxillary tuber and zygomatic arch which were absent in the studied specimens. Its insertion on the masseter fossa and the caudal and ventral portion of the mandible was not observed. A mixture of adipose tissue and muscle fibers on the caudolateral aspect of the body of the mandible was observed. The cheek area was vestigial and so the buccinator muscle (oral part) and depressor of the lower lip were displaced rostrally under the lower lip (Figure 4, Figure 5, Figure 6 and Figure 7). The orbicularis oris muscle was absent. Medial to the temporomandibular joint, the pterygoid muscles were easily seen (Figure 3, Figure 4, Figure 5 and Figure 6). The mandible depressor muscles, digastric and mylohyoid were well developed. The digastric muscle insertion enlarges until the most latero-rostral sections of the mandible body (Figure 3, Figure 4, Figure 5, Figure 6, Figure 7 and Figure 8). Muscles were easy to differentiate in anatomical sections. CT showed them moderately hypoattenuated and in MR images, slightly hypointense.

The mandible was hyperattenuated in CT and hypointense in MRI. The mandibular fat showed some dark color in anatomical sections; it was slightly hyperattenuated in CT sections, slightly hyperintense in T1-weighted, and slightly hypointense in T2-weighted sequences (Figure 3, Figure 4, Figure 5 and Figure 6). The mandibular canal was observed patent and wide in the three specimens studied, as it is usual in odontocetes.

### 3.2. Rostrum (Snout)

Dorsal to the oral cavity and below melon the rostrum is observed. The mesethmoidal cartilage could be seen amongst vomer, maxillary, and incisive bones, being supported only on the groove of the vomer bone. This cartilage acts as an adhesive joining these bones to each other (Figure 2, Figure 3 and Figure 4). CT showed one of two infraorbital canals inside the incisive bone of sco1 (Figure 2(R1B)). In the anatomical section, under the lateral sublingual recess, only fat and striated muscular tissue was seen, instead of glandular tissue, and this tissue was seen as a slightly hyper/hypointense area depending on the MRI sequence used (Figure 3).

### 3.3. Melon

The first two-level sections of the snout showed this particular anatomical structure of cetaceans (Figure 2 and Figure 3). Insco1, the most rostral part of the melon could already be observed (Figure 2(R1E)). The melon was observed hyperintense in T2-weighted FSE sequence but was not detected in PET/SPECT/CT and T1-weighted SE sequence. In sco2, the melon was well appreciated in anatomical sections as well as hypointense structure in MRI sequences (Figure 2(R2A, F)). In sco3, the melon was seen as a large diffuse area in both CT and MRI (Figure 2(R3)).

**Figure 9:**
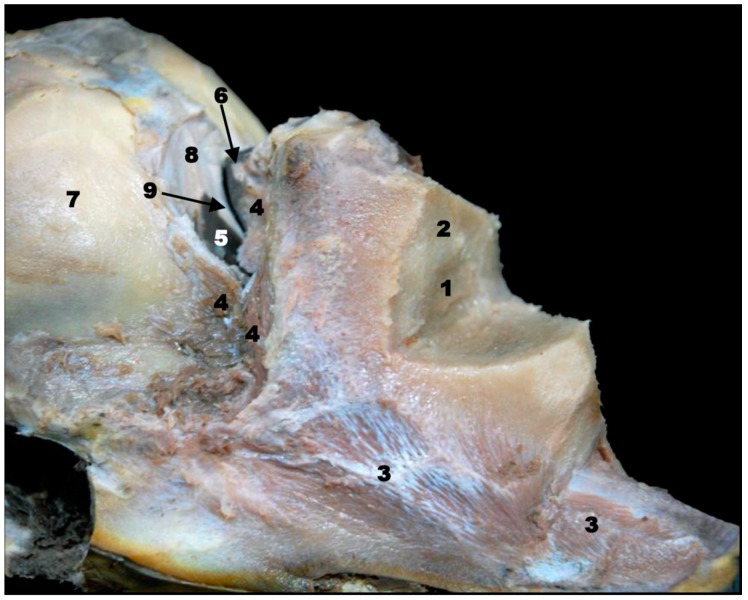
Deep head dissection made at the level of the nasal vestibule and melon of sco4. 1, Melon: nucleus; 2, melon: external fiber ring; 3, melon rostral muscles; 4, melon caudal muscles; 5, right nasal cavity (after removing nasal plug); 6, left nasal plug; 7, maxillary bone; 8, ethmoid bone; 9, nasal septum.

The caudal part of the melon encloses the nasal cavity. In sco1, it was slightly hyperintense in MRI sequences (Figure 3(R1D, E)) and it was not observed in PET/SPECT/CT (Figure 3(R1B)). In sco2, the anatomical section showed the white nucleus of the melon surrounded by connective tissue and muscles. However, only the nucleus was identified in CT and MRI sequences (Figure 3(R2)). In sco3, CT images showed the central nucleus of the melon as moderately hypoattenuated and its external fibrous ring as slightly hyperattenuated; in a similar way, the nucleus was hyperintense and the external fibrous ring hyper/hypointense depending on the MRI sequence (Figure 3(R3)). Dissection of the melon showed the nucleus and external fibrous ring as in CT and MRI (Figure 9).

### 3.4. Nasal Cavity and Pasanasal Sinuses

From the beginning of fetal development until birth, an opening was observed between the melon and the frontal bone. On external examination, the nostrils, also named the spiracle or blowhole, gives the common impression of an access to odd nasal vestibule. But two nostrils closed by a musculomembranous fold were observed. Under the nostrils was the dorsal part of the nasal cavity named the vestibule of the nose, which is divided in two (left and right) by the membranous part of the nasal septum. Between the nasal vestibules and nasal plugs, different diverticula were observed. In sco3, the nasal septum, nasal diverticula (hypoattenuated areas), and plugs were clearly visualized in both CT and MR sequences (Figure 4(R3)). Nasal plugs show a cartilaginous appearance (slightly hyperattenuated in CT and hypointense in MRI), with muscular fibers and mucosa (slightly hypoattenuated in CT, slightly hypointense in T1-weighted SE sequence, and hypointense in T2-weighted FSE sequence) (Figure 4 and Figure 5(R3)).

Under the nasal plugs (Figure 9), two nasal cavities were observed. The rostral boundary is formed by the maxillary bones, medially and caudally the vomer bone and perpendicular lamina of ethmoid bone dominate, respectively, while the lateral boundary is the pterygoid bone. The nasal septum is formed mainly by the vomer bone and by the perpendicular lamina of the ethmoid bone. In sco1, the vomer was observed hypoattenuated in CT and slightly hypointense in MR sequences and the mesethmoidal cartilage was seen hypoattenuated in CT and moderately hyperintense in MR sequences (Figure 4(R1)). In sco2, the vomer together with the ethmoid bone and mesethmoidal cartilage were identified in anatomical section. The vomer was hyperattenuated in CT and hypointense in all MR sequences. The mesethmoidal cartilage was clearly differentiated in all images except in MR sequences (Figure 4(R2)). In sco3, the vomer bone and mesethmoidal cartilage were hyperattenuated in CT, slightly hypointense in T1-weigthed MR SE sequence, and moderately hyperintense in T2-weigthed MR FSE sequence (Figure 4(R3)).

In all stages studied and with CT and MRI techniques, the maxillary sinus was observed as a small cavity within the maxillary bone. This sinus was observed in anatomical sections (sco2) filled with a heterogeneous substance observed in the fetal specimens examined in our study (Figure 3(R2A)).

Choanae are openings between the nasal cavity and the nasopharynx, and this space full of air was observed hypoattenuated in CT and hypointense in MRI sequences. In sco1, the mucosa and nasopharyngeal muscle were hypoattenuated in PET/SPECT/CT and slightly hyper/hypointense depending on the MRI sequence (Figure 5(R1)). Anatomical sections of sco2 showed mucosa and nasopharyngeal muscle with a small air space; in CT it was moderately hyperattenuated. MRI sequences showed it moderately hypointense or hypointense (Figure 5(R2)). In sco3, it could be seen as slightly hyperattenuated in CT and moderately hypointense in MRI sequences (Figure 5(R3)).

### 3.5. Orbit and Eyeball

CT images showed that the orbit is formed by an incomplete bony rim composed of a supraorbital part formed by the frontal bone; no infraorbital rim was observed. The rostral limit of the supraorbital rim is formed by the zygomatic and lacrimal bones. In sco1, a junction (synchondrosis) between the zygomatic and lacrimal bones was observed in PET/SPECT/CT sections (Figure 3(R1B)). Nevertheless, in sco2 (Figure 3(R2A)), anatomical sections showed both bones undergoing ossification (synostosis). Using CT, it was not possible to differentiate between these bones as the image was very hyperattenuated.

An oblique, thin temporal process of the zygomatic bone crossing under the periorbit and holding it was observed. CT showed the temporal process in sco1 with little bony density (hypoattenuated). Nevertheless, it was observed slightly hyperattenuated in sco2 and sco3 (Figure 3, Figure 4 and Figure 5).

The eyeball was observed in all MR sequences (Figure 5 and Figure 6) except in PET/SPECT/CT (Figure 5 and Figure 6(R1)). CT showed the lens hyperattenuated and hypointense in MR sequences (Figure 5(R2)). The tapetum lucidum was not appreciated in anatomical sections, but it was clearly seen in the dissection of sco4.

### 3.6. Central Nervous System

Brain hemispheres divided by the longitudinal brain fissure (hypointense in MR sequences) were observed in sco1 and sco2 but were not clear in sco3 (Figure 5). The sagittal dorsal sinus at the temporal level was also observed (Figure 6 and Figure 8). In sco1, the lateral ventricle could be distinguished in MR sequences, but it was more difficult to identify in sco2 and sco3, as well as in anatomical sections, CT, and MR sequences. The meninx was observed in both anatomical sections and MR sequences (Figure 8). The mesencephalic aqueduct was clearly seen in all MR sequences. In anatomical sections, the vestibulocochlear and facial nerves as well as the labyrinthine artery passing through the inner auditory meatus were identified (Figure 8(R2)); however, these anatomical structures were not observed in CT and MR sequences.

**Figure 10:**
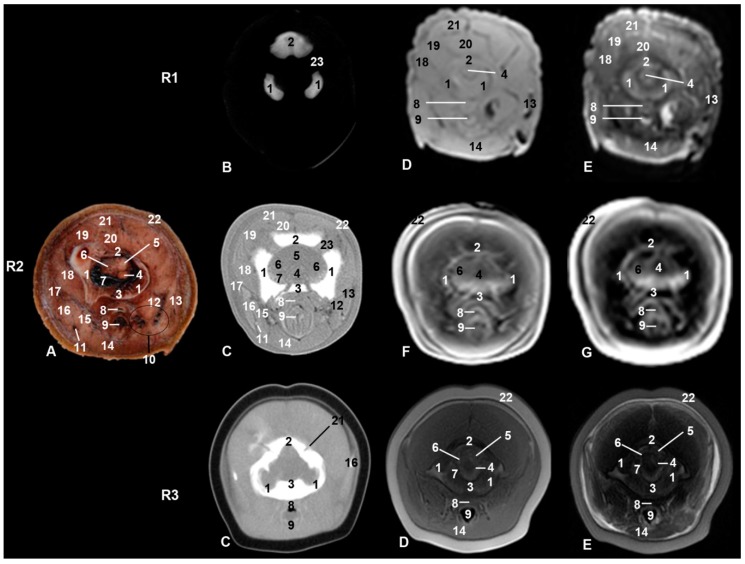
Representative transverse images made at the level of the occipital bone, cerebellum, and trunk of encephalon. Level VII. Images are oriented so that the left side of the head is to the right and dorsal is at the top. R1, sco1; R2, sco2; R3, sco3. (**A**) Anatomical section. (**B**) Vascular window PET/SPECT/CT image. (**C**) Soft-tissue window CT image. (**D**) T1-weighted SE sequence. (**E**) T2-weighted FSE sequence. (**F**) T1-weighted FFE sequence. (**G**) T1-weighted OOP GRE sequence. 1, Occipital bone: lateral part; 2, occipital bone: squamous part; 3, occipital bone: basilar part; 4, myelencephalon; 5, cerebellum: vermis; 6, cerebellum: cerebellar hemispheres; 7, subarachnoid space; 8 = esophagus; 9, laryngeal cavity: glottis; 10, vascular and nerve structures of the pharynx and larynx; 11, external jugular vein; 12, longus capitis muscle; 13, scapula; 14, sternohyoid and sternothyroid muscles; 15, cleidocephalic muscle: mastoid part; 16, sternocephalic muscle: mastoid part; 17, longissimus capitis muscle; 18, splenius capitis muscle; 19, semispinalis capitis muscle (digastric); 20, semispinalis capitis muscle (complex); 21, spinalis capitis muscle; 22, epidermis, dermis, and subcutaneous tissue; 23, fontanelles.

The cerebellar tentorium appeared hyperattenuated in CT and slightly hyperintense in both MR sequences (Figure 8(R2)). The vermis and cerebellar hemispheres were observed at the level of the cerebellar fossa held by the cerebellar tentorium (Figure 10(R2A)).

### 3.7. Ear

The petrous part of the temporal bone was sectioned at the bony labyrinth level, which contains the bony spiral canal, bony vestibule, and spiral canal of the cochlea. Although both petrous and tympanic parts of the temporal bone were seen with both diagnostic imaging techniques (CT and MRI), components such as the auditory ossicles of the middle ear, the spiral canal of the cochlea, and the bony vestibule were visible only in CT and in anatomical sections. The auditory ossicles of the middle ear were observed hypointense in MR sequences and hyperattenuated when using CT. Nevertheless, it was possible to see the malleus, incus, and stapes using anatomical sections and CT (Figure 8). The rudimentary cartilaginous part of the external acoustic meatus was a slightly hyperattenuated small area in sco3 using CT (Figure 8(R3B)) and was also observed in dissections of sco4 and sco5 (Figure 7). CT showed a fatty content slightly hypoattenuated in the tympanic cavity (Figure 8).

Connecting the tympanic cavity with the nasopharynx was an enlargement of the auditory tube which was observed only in sco2 and sco3 (Figure 6). In anatomical sections and in MR sequences, the pharynx was appreciated surrounding aditus laryngis (epiglottic and arytenoid cartilages), and the esophageal vestibule lies dorsally (Figure 8).

### 3.8. Larynx

Surrounding the laryngeal cartilages, the laryngopharynx was observed, as well as the hyoid apparatus in their relationship with the ear (Figure 8). In sco1, CT images of the hyoid bones were shown slightly hyperattenuated (Figure 8(R1)). In sco2 (Figure 8(R2)), the hyoid bones were larger, and in sco3 (Figure 8(R3)) were very hyperattenuated; in anatomical sections, the tympanohyoid bone was well appreciated (Figure 8(R2A)). Also observed in this area were the temporomandibular joint and the mandibular canal fat which was very close to the middle ear, making contact with the tympanic wall. The stylohyoid, basihyoid, and thyrohyoid bones were well observed using CT and dissections because they were ossified, while the epihyoid, ceratohyoid, and tympanohyoid bones were not seen because they still remain cartilaginous. The caudal tip of the thyrohyoid bone was not ossified at birth.

### 3.9. Cranial Cavity

Fontanelles were wide in sco1 (Figure 8(R1)), closing in sco2 (Figure 8(R2)) and almost closed in sco3. CT in both fetuses showed clearly the fontanelles. Bones of the cranial cavity were analyzed mainly using CT and anatomical sections, since MR sequences showed bones as hypointense in all cases.

The occipital bone has three parts: basilar, lateral, and squamous. The basilar part was not observed in sco1 at this level section (Figure 10(R1)) but fontanelles were clearly seen. The basilar part was observed in sco2 (Figure 10(R2)). In sco3 (Figure 10(R3)) fontanelles are closing, though the bones surrounding the foramen magnum are not totally ossified.

## 4. Discussion

### 4.1. Anatomical and Functional Considerations

#### 4.1.1. Oral Cavity

Comparing our study’s anatomical sections and PET/SPECT/CT images, we observed that in the fetus and newborn, the teeth of the rostral alveoli (equivalent to incisive teeth) erupt later than the more caudal alveoli (equivalent to premolars and molars). This would have a functional application in the lactation (perinatal period), where the rostral teeth erupting afterwards would to serve to help to suction milk and at the same time to hold the mother’s nipple without harming it, thanks to the fact that the teeth are not yet completely formed (Figure 1, Figure 2, Figure 3, Figure 4 and Figure 7). Odontocetes are (eu)homodont with conical teeth without a complete root, polydont, monophyodont [27] and tecodont, isognathous with centric occlusion of both dentary archs and with prognatism even during fetal stages, and are designed to catch prey [28].

We were unable to find the masseter muscle in fetal, newborn, and adult striped dolphin specimens, though it has been described by some authors in the striped dolphin [8,11], in a juvenile common dolphin [9], and in a bottlenose dolphin [29,30]; images from these studies suggest that the papers are describing the buccinator (oral part) and depressor muscles of the lower lip. (Figure 2(R2A) and Figure 9). After performing dissection of the head muscles in an adult striped dolphin, we conclude that the muscle atrophies, finding only remnants of adipose tissue and muscle fibers in its anatomical position (Figure 7). The origin of this muscle (mandible elevator) in domestic mammals extends from the maxillary tuber or facial crest to approximately the middle of the zygomatic arch, the two latter structures being absent in the pre- and perinatal studied specimens. Only a very thin temporal process of the zygomatic bone (jugal bone) is joined to the temporal bone by a symphysis, making it an unsuitable location for the attachment of a strong muscle, which acts to close the mandibles (Figure 3, Figure 4 and Figure 5). Also, the insertion in domestic animals is the masseteric fossa and medially on the ramus of the mandible [31,32], and the masseteric fossa is absent in odontocetes but not totally in mysticetes. Functionally, the masseter muscle is an extremely powerful muscle of mastication, which varies among species in terms of its topography [32]. Odontocetes have lost this feature throughout evolution, because most of the cetaceans, with the remarkable exception of orcas [33] swallow their prey intact without chewing. Reference [30] described a residual masseter muscle in odontocetes along with a more developed temporal muscle. In addition, mysticetes catch the krill between the whalebone and raise the mandibles full of weight (mostly water) to filter it (Humpback whales [34] and rorquals [35]). The masseter muscle was also described by [19] in the boreal whale fetus and by [36] in the Minke whale.

In this study, salivary glands and lymphatic nodules were not observed in either the fetal or the newborn dolphin heads. Under the lateral sublingual recess and folds (monostomatic and polystomatic), sublingual salivary glands were not observed, only a mass of striated muscle fibers and adipose tissue (Figure 2 and Figure 3). During dissection of the head and neck of an adult striped dolphin, only the superficial cervical lymphatic nodes were identified after histological analysis. This is logical in homodont animals, which swallow whole prey. Reference [30] showed the absence of organized salivary glands in dolphins. These same authors also describe lymphatic nodules of head to be smaller in size (about 1 cm) and therefore difficult to observe. In whales, the lack of salivary glands is described as well as the persistence of its ducts [26]. Strangely enough, an adenocarcinoma affecting the microscopic lingual salivary glands has been described in Beluga whales [37].

#### 4.1.2. Rostrum (Snout)

Telescoping refers to the overlap of the incisive and maxilla bones and the retraction of nasal bones on top of the frontal bone, as well as to the reduction of the temporal fossa and the rostral displacement of some muscles [3,12,28]. However, in the dolphin’s snout, there is a link between the vomer, maxilla, and incisive bones. The mesethmoid cartilage serves as an anti-concussive structure. The cartilage appears hypoattenuated in sco1 CT but moderately hyperattenuated in both sco1 and sco2. It does not show more signal intensity in T1-weighted SE sequence as described by [12], except in sco1. In the domestic mammal nose, the nasal septum cartilage extends rostrally to the nasal openings, but in cetaceans the nasal cavity and snout are at different levels due to telescoping. The snout tip has three functions in odontocetes: tactile (protopatic), offensive (together with tip of mandible), and, as a consequence of the mesethmoid cartilage joining these three bones, a shock absorber [8].

#### 4.1.3. Melon

The rostral muscles are inserted in the fibrous external area of the melon in the Tursiops fetus [11] and are called maxilonasolabialis muscles in adult specimens of striped dolphin [8], in the common dolphin [12], and in bottlenose dolphin [38]. These muscles are present in the beluga whale [39]. In our study, topographically, these muscles were located ventrolateral to the melon and belong to the residual group of the facial neuromuscular system [31,32,38] (Figure 2 and Figure 3).

#### 4.1.4. Nasal Cavity and Paranasal Sinuses

After emerging from water, the dolphin exhales air full of CO_2_ and steam through the nasal openings, nostrils. Surrounding the nostrils are striated muscle fibers opening and closing the nasal openings, acting as sphincter muscles. In our study, we could not clearly identify this group of muscles as they are mixed with melon muscles (Figure 4). In anatomical sections and MRI in a common dolphin, [9] identified the musculature of external sacs system and [40] specially studied them, which concluded that these muscles originate from the residual facial neuromuscular group, for example, orbicularis oris, levator nasolabialis, and the levator of the upper lip.

On dissecting the nasal vestibule in a fetal specimen, we observed that both nostrils have a common area under a musculomembranous sphincter, but ventrocaudally the membranous part of the nasal septum separates the left and right nasal vestibules. Reference [12] stated that the superficial blowhole emerges into an unpaired vestibulum. Ventral to the nostrils, nasal vestibules with several air sacs (diverticula) [41] were observed in the studied specimens (Figure 4 and Figure 5). The nasal vestibule is the rostral part of the nasal cavity, related to the nasal diverticulum (horses) and lined by stratified squamous epithelium [24]. This epithelium is also referred by [30] as “similar to the epidermis of the animal’s back” as in the equine vestibule. The nasal diverticulum has been described in horses as a cutaneous blind pouch inside the vestibule. The alar fold is a mucosa supported by the medial accessory cartilage covered dorsally by lateral nasal muscle [24,31,32] in the nasal vestibule in horses. In dolphins, the nasal plug, alar fold, and the nasal vestibule have been modified for sound generation and as a water reservoir. Different authors have named the nasal diverticula as sacs or air sacs or sinuses. References [8,42] described diverticula as vestibular, tubular, and premaxillary sacs. Reference [43] added the nasofrontal, accessory, and connecting air sacs and shows the premaxillary sac under the nasal plug and inside the nasal cavity [8,44]. Reference [12] mentioned three vestibular sacs. Reference [41] added the pterygoid and laryngeal air sacs. Several authors [45,46,47,48] explained that the dolphin head shows a very complex structure with unique air sacs and special sound-conducting fats. At the same time, Reference [49] claimed that no paranasal air sinuses form within the skull either prenatally or postnatally. In addition, [8] described the presence of pterygoid and maxillary sinuses with a heterogeneous substance in sections III and IV as we found in sco2 (Figure 3(R2A)). These sinuses may be non-functional. In contrast, references [26,41] describe only the pterygoid sinus (filled with a heterogeneous substance) in CT and anatomical sections in the bottlenose dolphin, whereas the maxillary sinus was the only sinus detected in our study. The paranasal sinuses heat the air and decrease the skull weight in domestic mammals. In cetaceans, moving in a less gravid environment could lead to regression of these cavities. Reference [1] said that there are no paranasal sinuses in *Tursiops truncatus*, and that in the remaining odontocetes, the maxillary sinus is described (Figure 3) (probably without functionality), though it is not well studied in whales. On the other hand, [10] described the ethmoidal sinus and [50] the pterygoid sinuses, which we have not observed in our specimens studied. We have located in striped dolphin some small orifices in the frontal wall of the nasal skull that clearly connect the nasal cavity with the maxillary sinus; however, we do not know if nasal mucosa closes these orifices completely or are vascular nutrition orifices.

The nasal cavities are symmetrical and present a similar diameter compared to those of domestic mammals [31,32]. In odontocetes, there is a skull asymmetry where structures of one side (fluctuating, that means, in one individual a structure on the right side is larger, whereas in another individual from the same species, a structure on the left side is larger) are consistently larger than those on the other [51]. This asymmetry is assumed to be based on biosonar production and reception, but it has been suggested that the asymmetry is directly proportional to the prey size, so through evolution, the larynx and the hyoid apparatus have been pushed to the left in order to extend the pharyngeal canal and allow larger prey to be ingested [52].. The ethmoid bone shows small foramina opening into the nasal mucosa equivalent to the cribriform plate observed in domestic mammals. Nasal conchae and ethmoturbinates are not observed as are found in other domestic mammals [1]. These same authors state that the ethmoid bone in cetaceans forms the dorsocaudal wall of the nasal cavity. In a striped dolphin newborn skull, we observed an ethmoid bone like the cribriform plate in domestic mammals; therefore, we believe it is possible that olfactory mucosa may be present. We agree with [30] who described the presence of “minute foramina” in very young individuals, reminiscent of the ethmoid bone cribriform plate. Reference [28] also talked about a certain olfactory capacity in cetaceans. On the other hand, [1] claims that, according to [53], in Tursiops and other odontocetes the cribriform plate appears unperforated. We did not observed the vomeronasal organ in our anatomical sections or MR sequences under the nasal mucosa and vomer bone (Figure 4).

Reference [28] described the pterygoid bone as enlarged and much pneumatized at the ventral part of nasal cavities (choanae) in beaked whales, showing medial and lateral bone laminae. According to [54], some cetaceans lost the lateral bone lamina and this lamina is replaced by the tendinous lamina, which holds the palate muscles.

#### 4.1.5. Orbit and Eyeball

Reference [55] described an early stage of development (8–15 cm long.) in the pantropical spotted dolphin fetus, noting the lacrimal bone and later (21–22.5 cm long) the zygomatic (jugal) bone well-ossified and rostrally fused firmly with the lacrimal bone. However, in our study using PET/SPECT/CT, it is possible to observe in sco1, ossification centers which are not fused at this early stage (Figure 3). According to [1,12,30], in odontocetes (except beaked whales), the lacrimal and zygomatic (jugal) bones are fused forming the lacrimozygomatic (lacrimojugal) bone, a fact that we have confirmed in this study. The microtomographies performed on sco1 reveals that these two bones show a slightly hypoattenuated contact line indicating that it is a synchondrosis that will become a synostosis, meaning that they will be fused. As in domestic mammals, the lacrimal bone is placed lateral with respect to the zygomatic bone. The caudal projection of the zygomatic bone, the temporal process (zygomatic arch) [30], holds the periorbit and establishes a symphysis with the orbital surface of the temporal bone. The temporal process of the zygomatic bone in domestic mammals forms the infraorbital border together with the periorbit (fibrous fascia), which holds the eyeball and periorbit. In odontocetes, there is no bony infraorbital border and the only structure running under the ventral eyelid is the facial nerve. In this study, the temporal process of the zygomatic bone holding the periorbit at its middle point was observed in dissections and anatomical sections (Figure 3, Figure 4, Figure 5, Figure 6 and Figure 7B).

#### 4.1.6. Central Nervous System

MR sequences of the brain of sco1 begin to differentiate the diencephalon and telencephalon, though the trunk of the encephalon (medulla oblongata, pons, and mesencephalon) and cerebellum are less defined. In sco2, the different parts of the trunk of the encephalon are better defined, except for the cerebellum. In addition, we have observed the cerebellum and brain hemisphere as described in perinatal dolphins [12]. The lateral ventricles and the mesencephalic aqueduct were appreciated in sco1, sco2, and sco3. We have not observed the fourth ventricle using MR sequences either in sco1 and sco2 or in sco3, even though [15] described it in a sub-adult specimen using sagittal MR sequences (Figure 5, Figure 6, Figure 7 and Figure 10). Both developmental fetal stages allow us to differentiate the cerebellar tentorium as hyperintense in MR sequences. Therefore, we have observed that the tentorium cerebelli starts ossification during the fetal stage until the process is complete in adult odontocetes, unlike domestic mammals, where it remains membranous, except in cats [31,32]. We suggest the functional explanation for this relates to both species needing a well-held cerebellum due to their activity during swimming (locomotion), jumping, climbing, etc. Fetal and adult dolphin cerebellar hemispheres are situated laterally and parallel staying almost at the same level of the ventral surface of the encephalon trunk, as it can be observed in the 3D reconstruction of a common dolphin fetal brain [14].

#### 4.1.7. Ear

The external acoustic meatus and the auricular cartilage have their origin from the projection of the first two pharyngeal arches toward the pharyngeal cleft which forms the external ear [56]. The external ear is absent in cetaceans and only remains as a small epidermal depression, barely visible in fetal specimens and newborns. At subcutaneous levels, cartilaginous nests of the external acoustic meatus appear hyperattenuated in CT in sco3. The middle ear or tympanic cavity is formed by the dilatation of the bottom of the first pharyngeal pouch and the remainder of the pouch forms the auditory tube, while the inner ear has an ectodermal origin [57]. We see both middle and inner ears in anatomical sections and tomographies as hyperattenuated (Figure 8), compact bone with thin bony walls in the fetus, with the walls thicker and hyperattenuated at the prenatal stage in agreement with [12]. In our study, MR sequences shows as hypointense the middle and inner ear walls while in our three MR sequences the hyperintense areas correspond with the ossicles and tympanic cavity described by [12] in MR T1-weighted sequence.

At the tympanic cavity, we have observed a semi-open musculotubal canal in sco2 [1], whose opening is the tympanic orifice located in the carotid or rostral wall of middle ear. This continues with the auditory tube to its opening at the pharyngeal orifice in the nasopharynx (Figure 6).

The auditory tube in sco4 could be observed in dissections as a mucosal-bony dilated space close to the perpendicular lamina of the palatine bone, extending to the pharyngeal orifice of auditory tube (eustachian notch) [1] where it opens into the nasopharynx by the pharyngeal orifice of the auditory tube. We agree with [58] about the presence of a maxillary sinus and also about the existence of a connection with the middle ear (auditory tube that we observed dilated), but not about the set of aerial sacs (premaxillary, vestibular, and nasofrontal) [30].

In our fetal study, we have just observed in CT some hypoattenuated areas (air cavities) close to the musculotubal canal of the middle ear, which could be a “pseudo-diverticulum” similar to the horse “guttural pouch” or a pharyngeal diverticulum of the auditory tube [30,31]. That would form a rudimentary osteomucomembranous space or guttural pouch, also placed in the cetaceans under the base of the cranium, connecting the tympanic cavity with the pharyngeal orifice of the auditory tube inside the nasopharynx (Figure 6).

#### 4.1.8. Larynx

During feeding, cetaceans need the hyoid apparatus to expand, be flexible and extensible, and to project the larynx caudally (retraction) and the tongue ventrally (depression) in order to allow food to pass into the esophagus. If the hyoid apparatus was rigid, fractures could occur during feeding. In odontocetes and eschrichtiids (gray whales), increased tongue musculature and enlarged hyoids allow grasping and/or lingual depression to generate intraoral suction for prey ingestion [33]. On the other hand, balaenopterids need the mandible to be open to 90° so that the oral cavity holds up to 60 m^3^ of water, so these specialized mechanisms also affect the anatomical model of the mandible and maxilla [59], as happens, for instance, in the humpback whale [60].

Nevertheless, in fetal specimens, we have observed that the entrance to the larynx (larynx peak) is very circumscribed and is formed by the rostral tips of the epiglottic and arytenoid cartilages. Both cartilages are very enlarged and oriented dorsorostrally toward the choanae. The arytenoid cartilages should not be described as cuneiform cartilages [12,29,30] and perhaps as corniculated tubercles [31]. The cuneiform cartilages are only described in the horse epiglottic cartilage and the dog’s arytenoid cartilages. The corniculate tubercules are indeed mucosal eminences formed by the corniculated process of the arytenoid cartilages [24,31,32], a feature not observed in the specimens studied (Figure 8).

In the dolphin anatomical section and dissection, we have observed a cartilaginous tympanohyoid (Figure 8(R2A)) connecting the stylohyoid bone with the tympanic part of the temporal bone, placed externally on the mastoid wall of the middle ear [1,61]. Its function is to hold the tongue root and the larynx to the base of cranium as in the domestic mammals [31] while the thyrohyoid attaches to the paracondylar process of the lateral part of the occipital bone—paraoccipital process of the cranial basal bones according to [30], which is different from domestic mammals, where the thyrohyoid attaches to the rostral horn of thyroid cartilage of the larynx [31]. In addition, in the dolphins studied, the caudal tip of the thyrohyoid does not become ossified at birth, thus remaining in the adult as in gray whale [62].

#### 4.1.9. Cranial Cavity

Three fontanelles were observed in our odontocetes fetus studied: occipital, frontal, and mastoid [1] (the last one less clear) and are confused in *Stenella attenuata* [55]. Studies in mysticetes could not add more information for comparison with dolphins [63].

## 5. Conclusions

Fetal anatomical sections have been very important to ensure that certain anatomical structures were correctly identified, but we needed the dissections to confirm the presence of these structures. CT was used to identify the bony and cartilaginous features of both the fetal and newborn specimens. On the other hand, different MRI sequences were used to recognize and differentiate visceral structures, which will help clinicians to diagnose different pathologies in the dolphin’s head region.

We have also observed that rostral teeth erupt after lactation and the perinatal period helping suctioning milk and to protect the mother’s nipples. Moreover, we have observed the absence or atrophy of the masseter muscle from fetal to adult stage in striped dolphins, mainly due to the presence of adipose tissue mixed with random muscle fibers in its anatomical position and because they swallow their prey.

No major salivary glands and lymphatic nodes were observed during developmental stages in dolphin heads, only a mixed mass of muscle fibers and fat.

A maxillary sinus has been observed filled with a heterogeneous content in our study from fetal to perinatal stage and could be non-functional.

The fusion between the lacrimal and the zygomatic bones was observed in the early fetal specimen. The temporal process of the zygomatic bone holding the periorbit in fetal dolphins has been described.

Finally, we can conclude that a “pseudo-diverticulum” similar to the “guttural pouch” connecting the tympanic cavity (middle ear) with the nasopharynx was observed in fetal anatomical sections.

## Figures and Tables

**Table 1:** Fetal specimens of striped dolphin used in this study.

Stranding Reference and Study Code	Sex, Length, Weight and Estimated Gestation Time [7,25,26]	Anatomical and Imaging Diagnostic Techniques	Preservation Techniques
SCOG	Female fetus, 32.5 cm, 508 g, 4.5 months	MRI, PET/SPECT/CT,	Fixation: formaldehyde 10%
CEMMA			
sco1			
SCOCE1	Male fetus, 48 cm, 1.535 kg, 7 months	MRI, CT, anatomical head sections	Fixation: formaldehyde 10%
CECAM			
sco2			
SCOCE2	Female newborn, 95 cm, 10.84 kg	MRI, CT	Freezing × 20 °C
CECAM			
sco3			
SCOCE3	Male newborn, 85 cm, 9.2 kg	Head dissection	Fixation: formaldehyde 10%
CECAM			
sco4			
SCOMU	Adult female, 1.91 cm, 63.65 kg	Head dissection	Embalming: formaldehyde, glycerine, isopropyl alcohol, phenol
CRFS			
sco5			

*SCOG*: *Stenella coeruleoalba* from Pontevedra, Spain; *SCOCE*: *S. coeruleoalba* from Ceuta, Spain; *SCOMU*: *S. coeruleoalba* from Murcia, Spain; MRI: Magnetic resonance imaging; CT: Computed Tomography, *CEMMA*: Coordinator Center for the study of the marine mammals, Galicia; *CECAM*: Center for the study and conservation of marine animals, Ceuta; *CRFS*: Wildlife rehabilitation Center, Murcia.
